# Spinal Arachnoid Web

**DOI:** 10.5811/cpcem.7189

**Published:** 2024-06-03

**Authors:** Maiya Smith, Morgan Ketterling, Alexander Gallaer, Rowan Kelner, Christine Raps, Allison M. Beaulieu

**Affiliations:** University of Utah, Department of Emergency Medicine, Salt Lake City, Utah

**Keywords:** *spinal arachnoid web*, *neurology*

## Abstract

**Case Presentation:**

We describe a case of a 57-year-old male with multiple medical comorbidities who presented to the emergency department with a two-week history of upper back pain with associated numbness. Physical exam demonstrated sensory loss in a bilateral third and fourth thoracic dermatome distribution. The diagnosis of spinal arachnoid web was made based on neurological exam and imaging findings.

**Discussion:**

Spinal arachnoid web is a rare diagnosis, but consideration is important, as early recognition and surgical intervention can resolve symptoms and prevent worsening neurological sequelae.

CPC-EM Capsule What do we already know about this clinical entity?

*Spinal arachnoid web is a rare diagnosis that presents with numbness and other neurologic sequelae secondary to thickening of the arachnoid and compression of spinal cord.*

What is the major impact of the image(s)?

*
Thickening of the arachnoid can be seen on magnetic resonance imaging as a “scalpel sign,” aiding in this ultimately surgical diagnosis.*

How might this improve emergency medicine practice?

*This rare disease is reversible when treated early. Physician awareness and use of imaging will aid in diagnosis and prevention of neurologic morbidity.*

## CASE PRESENTATION

A 57-year-old male with history of type II diabetes mellitus, renal transplant, coronary artery disease, and hypertension presented to the emergency department (ED) for numbness in his chest for two weeks, with associated upper back pain radiating to his chest bilaterally, and shortness of breath. He presented to an outpatient clinic for similar complaints one day prior and was started on a four-day course of prednisone for presumed pleurisy.

On examination in the ED, the patient was found to have decreased sensation in his third and fourth thoracic dermatome in a band-like distribution without additional neurologic deficits or skin findings. Magnetic resonance imaging (MRI) of the cervical and thoracic spine were obtained, showing a dorsal spinal arachnoid web (SAW) with slight compression of the spinal cord located at the third and fourth thoracic levels (Image, Video).

**Image.**
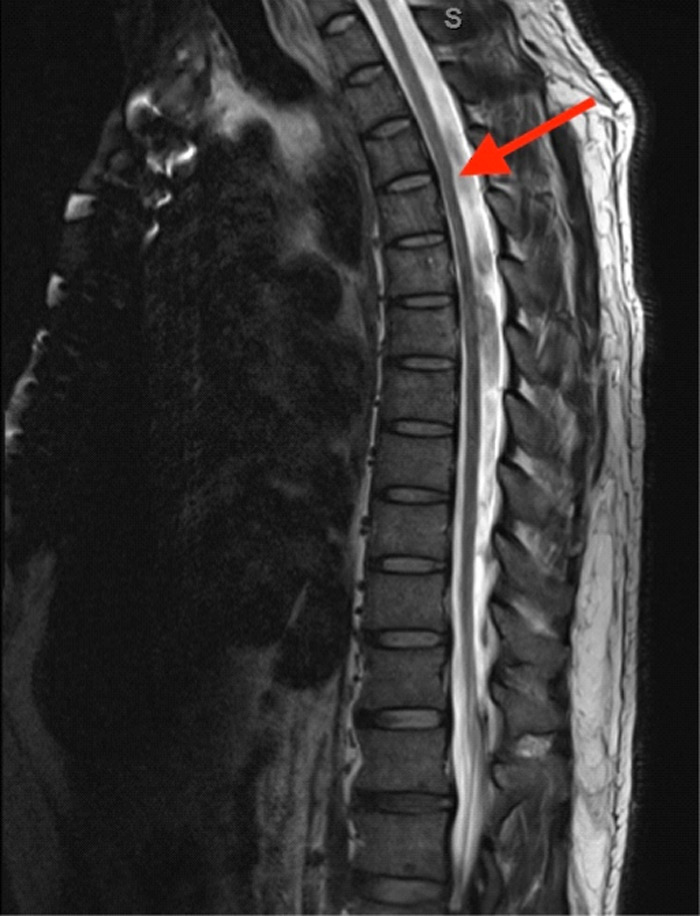
Magnetic resonance imaging with and without contrast showing focal anterior displacement of the thoracic spinal cord at the third and fourth thoracic levels (arrow). In the setting of a prominent dorsal subarachnoid space with altered cerebrospinal fluid flow dynamics, the findings demonstrate a dorsal arachnoid web.

Neurosurgery was consulted, evaluated the patient and reviewed imaging. No surgical intervention was offered at that time due to the patient’s significant comorbidities and moderate symptoms. He was discharged home from the ED with recommended close outpatient follow-up.

## DISCUSSION

Spinal arachnoid web is a rare diagnosis. Within the meninges, there are three layers: the dura, arachnoid, and pia. The arachnoid is a thin membrane between the dura and pia that adheres to the brain and spinal cord.^1^ A SAW specifically refers to a focal thickening of the arachnoid, typically in the thoracic spine, which causes compression of the spinal cord and interferes with the free flow of spinal fluid within the dorsal subarachnoid space. It is thought that SAW represents a variant of arachnoid cyst formation. While this patient did not have radiographic evidence of syringomyelia, SAW is typically associated with syringomyelia and does not seem to be associated with trauma, hemorrhage, or inflammation.^2^ Presenting symptoms include back pain, upper/lower extremity weakness, and numbness.^3^

Imaging includes MRI or computed tomography myelography and often demonstrates a “scalpel sign” deformity at the site of the SAW, representing the focal dorsal indentation caused by the web, reminiscent of the pointed edge of a scalpel.^3^ However, the only definitive diagnosis for SAW is through surgical confirmation.

Spinal arachnoid web is likely under-recognized and under-diagnosed given its rarity. Diagnosis usually takes years, and treatment involves surgical lysis of the arachnoid band.^2^ ^–^ ^4^ Surgical intervention can completely resolve symptoms.^2^ Failure to diagnose SAW may result in worsening spinal cord function and neurologic function. Patients who have progressively worsening pain, paresthesia, or weakness in a dermatomal distribution without trauma or prior neurosurgical intervention should prompt consideration of this diagnosis. Emergency physicians need to be aware of this rare diagnosis given its possibly irreversible neurological sequelae including pain, numbness, weakness, and paralysis.^2^ ^–^ ^4^

## Supplementary Information

Video. Magnetic resonance imaging with and without contrast demonstrating the “scalpel sign” seen at the third and fourth thoracic levels (arrow) due to the spinal arachnoid web.
